# Comparative analysis of distinctive transcriptome profiles with biochemical evidence in bisphenol S- and benzo[*a*]pyrene-exposed liver tissues of the olive flounder *Paralichthys olivaceus*

**DOI:** 10.1371/journal.pone.0196425

**Published:** 2018-05-01

**Authors:** Jee-Hyun Jung, Young-Sun Moon, Bo-Mi Kim, Young-Mi Lee, Moonkoo Kim, Jae-Sung Rhee

**Affiliations:** 1 Oil and POPs Research Group, Korea Institute of Ocean Science and Technology, Geoje, South Korea; 2 Department of Marine Environmental Science, Korea University of Science and Technology, Daejeon, South Korea; 3 Unit of Polar Genomics, Korea Polar Research Institute, Incheon, South Korea; 4 Department of Life Science, College of Natural Sciences, Sangmyung University, Seoul, South Korea; 5 Department of Marine Science, College of Natural Sciences, Incheon National University, Incheon, South Korea; 6 Research Institute of Basic Sciences, Incheon National University, Incheon, South Korea; University of Missouri Columbia, UNITED STATES

## Abstract

Flounder is a promising model species for environmental monitoring of coastal regions. To assess the usefulness of liver transcriptome profiling, juvenile olive flounder *Paralichthys olivaceus* were exposed to two pollutants, bisphenol S (BPS) and benzo[*a*]pyrene (BaP), which have different chemical characteristics and have distinct modes of metabolic action in teleost. Six hours after intraperitoneal injection with BPS (50 mg/kg bw) or BaP (20 mg/kg bw), liver transcriptomes were analyzed using the Illumina Hiseq 3000 platform. Interestingly, the transcriptome was highly sensitive and was distinctively expressed in response to each chemical. The primary effect of BPS was significantly increased transcription of egg process and vitellogenesis related genes, including vitellogenins (*vtg1*, *vtg2*), zona pellucida sperm-binding proteins (*zp3*, *zp4*), and estrogen receptors (*erα*, *erβ*), with increases in plasma 17β-estradiol (E2) and vitellogenin (VTG) concentrations. Following BaP treatment, detoxification- and biotransformation-related genes such as *cyp1a1* and UDP-glucuronosyltransferase (*ugt1a1*) were significantly increased, with an increase in EROD activity. In both transcriptomes, mRNA expression of genes involved in antioxidant defense systems was increased, while genes involved in innate immunity were decreased upon BPS or BaP exposure with a decrease in complement activity. This study provides useful insight into the chemical-specific hepatic transcriptional response of *P*. *olivaceus* and suggests a basis for further studies examining biomarker application of liver transcriptomes for environmental pollution.

## Introduction

Flatfish are bottom-dwelling fishes that spend most of their life cycle on the bottom of estuaries and coastal regions. Thus, flatfish are acutely or chronically exposed to a wide range of environmental pollutants derived from agricultural and industrial wastes, municipal sewage, and human activity [1]. Due to their benthic habitats and wide-ranging geographical distributions, flatfish could be potential indicators of water and sediment quality, using their molecular, biochemical, and physiological sensitivity. Numerous biomarkers have been continuously developed to understand the potential effects of environmental pollutants on flatfish in their role as sentinels [2–5]. The capacity of a biomarker to respond may be prolonged or restricted by complex molecular pathways with combinations of certain signal cascades, as numerous aquatic pollutants are released in different forms with distinct modes of action in waterbodies. Particularly, biochemical markers, including molecular responses, have been identified as powerful and cost-effective approaches to obtain information on the status of the environment and the effects of pollution on resident species, including flatfish [5–9]. However, the accessibility of genomic resources (e.g., whole genome and transcriptome) is limited in most marine fish, even though gene expression profiling has been widely applied to predict the potential toxicity of various chemical compounds and to elucidate the underlying molecular mechanisms.

The olive flounder *Paralichthys olivaceus* (also known as the Japanese flounder or bastard halibut) used in this study is one of the most cosmopolitan aquaculture species in east and south Asia, including South Korea, Japan, and China. *P*. *olivaceus* is desirable for commercial culture and was subsequently introduced in many Asia countries for aquaculture production. In 2011, the catch of wild *P*. *olivaceus* was approximately 4,600 metric tons and its aquaculture production was over 40,000 metric tons [10]. One of the most important steps in developing a model species for environmental monitoring is the availability of genomic information. Genomic platforms have been successfully applied to understand diverse molecular and physiological characteristics of *P*. *olivaceus* [11–14]. The recently published whole genome sequence of *P*. *olivaceus* provides a unique advantage for genomic application among flatfish [15]. Despite these advantages, application of genomic information of *P*. *olivaceus* in marine environmental monitoring has received little attention to date.

The primary objective of the present study was to test whether the transcriptome profiling of the olive flounder could be usefully applied for the monitoring of marine pollutants. Because the sensitivity of signal pathways is very different for chemicals, two distinct chemical pollutants were employed in this study. Bisphenol S (BPS) is a structural analog of bisphenol A (BPA) and is used as an alternative for BPA due to increasing concerns over the endocrine disrupting potential of BPA [16]. However, many *in vivo* and *in vitro* studies have suggested that BPS is as hormonally active as BPA with endocrine disrupting effects [17]. Benzo[*a*]pyrene (BaP) is a polycyclic aromatic hydrocarbon (PAH) and a ubiquitous marine pollutant derived from numerous human activities [18]. Adverse effects of BPA (e.g., carcinogenicity, genotoxicity, immunotoxicity, and neurotoxicity) and its biotransformation mechanism have been extensively reviewed [19, 20]. Biotransformation of xenobiotics and drug metabolism (e.g., oxidation, reduction, hydrolysis) occurs by the action of one or more enzymes in the liver tissue [21, 22]. In particular, both chemicals have consistently been found up to microgram level in the sediments and waterbodies of the coastal regions of south Asia, which are known to be among the olive flounders’ habitats [23–26]. Therefore, we comparatively analyzed BPS- and BaP-exposed liver tissues of *P*. *olivaceus* to consider its hepatic transcriptome as a promising tool for pollution monitoring of coastal regions.

## Materials and methods

### Fish culture and chemical exposure

All animal handling and experimental procedures were approved by the Animal Welfare Ethical Committee and Animal Experimental Ethics Committee of Korea Institute of Ocean Science and Technology (KIOST, South Korea). One-year-old juvenile olive flounder individuals weighing 497 ± 13.85 g (mean ± S.D.) and 21 ± 2.42 cm (mean ± S.D.) in length were purchased from a local fish farm in Geoje, Korea. The fish were placed in a 100-t tank supplied with circulating filtered seawater, and acclimated for two weeks. Photoperiod was maintained at 12/12 h light/dark condition. Water conditions were maintained at 18 ± 0.58°C, pH 7.7–8.0, and 87% oxygen saturation. Control fish were injected with dimethyl sulfoxide (DMSO, Sigma Aldrich, St Louis, MO, USA). Control (2 fish per control group) and each experimental group fish (3 fish per group) were intraperitoneally injected with bisphenol S (BPS, Sigma Aldrich, St Louis, MO, USA) at a concentration of 50 mg/kg body weight (bw) or benzo[*a*]pyrene (BaP, Sigma Aldrich, St Louis, MO, USA) at a concentration of 20 mg/kg bw. Three fish from each tank were anesthetized by immersion in buffered tricaine methanesulfonate (MS-222, 200 mg/L, Sigma Aldrich, St Louis, MO, USA) and subsampled 6 h after exposure. Liver tissues were frozen in liquid nitrogen and stored at -80°C for further analysis.

### Total RNA extraction and library construction

The entire procedure for Illumina RNA sequencing (RNA seq) was performed in Macrogen Inc. (Seoul, South Korea). A set of RNA samples for each chemical, comprised of three biological replicates, was not pooled. Each liver tissue was frozen in liquid nitrogen and homogenized with a glass pestle. Total RNA was extracted from individual olive flounder (two fish from control and three fish from BPS and BaP, respectively) using TRIzol^TM^ Reagent (Invitrogen, Carlsbad, CA, USA) according to the manufacturer’s instructions. DNA digestion was performed using DNase I (Sigma Aldrich, St Louis, MO, USA). Prior to Illumina RNA sequencing, total RNA quantity and quality were checked by analyzing the ratios A230/260 and A260/280 using a NanoDrop^®^ 2000 Spectrophotometer (Thermo Scientific, Wilmington, DE, USA) and an Agilant 2100 Bioanalyzer (Agilent, Böblingen, Germany) respectively. Samples satisfying the criteria of RNA integrity > 7.5 were used for library preparation.

Library construction was performed using Tureseq™ RNA sample prep Kit (Illumina, San Diego, CA, USA) at Macrogen Inc. (Seoul, South Korea) [27]. Briefly, the poly A-containing mRNA was purified using oligo dT-coated magnetic beads. The first strand of cDNA was synthesized using random hexamers and subsequently double-stranded cDNA was synthesized and purified [28]. After adaptor ligation, proper DNA fragments were selected and enriched to create the final cDNA library template. All libraries were sequenced by Illumina Hiseq 3000 with read length of 101 bp.

### *De novo* assembly, differentially expressed genes (DEGs) identification, and gene ontology (GO) analysis

Entire raw reads were cleaned with Trimmomatic by filtering with the following criteria: adaptor-only nucleotides, unpaired reads, empty nucleotides (N at the end of reads), short reads (< 59 bp), and low-quality nucleotides (reads containing more than 50% bases with Q-value ≤ 20) [29]. After quality control, large contigs were constructed using the *de novo* assembler Trinity (ver. 2.0.6). TransDecoder was used to identify candidate coding regions from the assembled transcripts and/or contigs (http://transdecoder.sourceforge.net). The candidate coding regions were used for BLAST analysis against the UniProt and the NCBI non-redundant (nr) protein database to evaluate sequence similarity to genes of other species using an E-value cutoff of 1e-06.

The abundance of each transcript was analyzed by direct alignment of each read to the control group assembly using the Bowtie 2 alignment tool (http://bowtie-bio.sourceforge.net/bowtie2) with the default parameters and RSEM method (RNA-Seq by Expectation-Maximization; http://deweylab.biostat.wisc.edu/rsem). The quantified number of mapped reads was normalized as fragments per kilobase of transcripts per million fragments mapped (FPKM) value. The false discovery rate (FDR) was used to predict the *P* value threshold for statistical analysis. The FPKM value of each sample was compared using the R package provided by Trinity platform. When the FDR value of a contig was less than 0.05 and its FPKM value was more than a two-fold change, the contig was considered to contain a differentially expressed gene (DEG). Hierarchical clustering analysis was employed in order to construct a heat map using MeV v.7.4 (Dana-Farber Cancer Institute, Boston, MA, USA) software. Gene Ontology (GO) analysis of the contigs was performed using the GOstats program (http://www.bioconductor.org/packages/3.3/bioc/html/GOstats) and Fisher’s Exact Test (*P* < 0.05), as implemented in the sequence annotation tool Blast2GO [30].

### Data deposition

All the raw data are available in the Sequencing Read Archive (SRA) of NCBI under the BioProject number PRJNA421418, with the accession number SRP126397 (SRX3459054- SRX3459061).

### Quantitative PCR validation

Total RNAs were reverse-transcribed into cDNA using a first-strand cDNA synthesis kit (Invitrogen, Carlsbad, CA, USA). The optical density at 260 nm and 280 nm (OD260/280) was approximately measured as 1.9, and the OD260/230 was between 1.8 and 1.9. The quantitative reverse transcription polymerase chain reaction (qRT-PCR) was performed using a two-step procedure. The *β-actin* gene was used as a reference control for real-time qRT-PCR. To quantify the mRNA expression level, we used the comparative CT method (2^-ΔΔCt^ method) with Roto-Gene Q (Qiagen, Hilden, Germany) according to the manufacturer’s instruction. mRNA expression for each gene was measured in triplicate. The specific primers for the qRT-PCR analysis were designed based on RNA-SEQ library using the ABI PRISM Primer Express software (Applied Biosystems, Darmstadt, Germany).

### EROD activity

The 7-ethoxyresorufin-O-deethylase (EROD) activity assay was conducted according to previous studies with slight modifications (e.g. buffer volume, employed basic instruments) [31]. The liver tissues of individual fish were homogenized in 0.25 M sucrose and centrifuged at 9,000 × *g* for 20 min at 4°C. To sample the microsomal fraction, the supernatant was transferred and centrifuged at 105,000 × *g* for 60 min at 4°C. Approximately 35 μg of microsomal fraction was transferred into the EROD buffer (0.1 M NaPO4, pH 7.6) with the addition of 7-ethoxyresorufin (ER) solution (0.4 mM ER in DMSO) and NADPH solution (10 mM NADPH in distilled water). The background subtraction was prepared with the same mixed buffer with the absence of the microsomal fraction. The resourufin production was measured with 530 nm excitation and 595 nm emission filters with a Varioskan Flash fluorometer (Thermo Fisher Scientific, Tewksbury, MA, USA). Results were represented as μmol min/mg protein.

### Plasma analysis

In our preliminary study, no significant changes in plasma 17β-estradiol (E2) or vitellogenin (VTG) were observed at 6 h after the injections of both chemicals. Thus, we extended our analysis to 24 h after the injection.

The E2 levels in the plasma were quantified by a standard ethyl ether extraction method using a commercial Estradiol ELISA kit (Cayman Chemical Company, Michigan, USA). Approximately 1 ml of blood was taken from the caudal vein of individual anesthetized olive flounders (n = 5) with a heparin syringe. The whole blood sample was centrifuged at 3,000 × *g* for 5 min and the supernatant was transferred for E2 analysis. The extract was centrifuged at 2,000 × *g* for 30 min and the ether phase was removed. The samples were dried using a centrifugal evaporator. The intra-assay coefficients of variance for both the fish were < 10%. The E2 concentration was measured with a Varioskan Flash spectrophotometer (Thermo Fisher Scientific, Tewksbury, MA, USA) at 420 nm, and was expressed as pg/mL.

The concentration of VTG in the plasma samples was determined using a commercially available enzyme-linked immunosorbent assay (ELISA) kit for VTG (MyBioSource Inc, San Diego, USA), following the manufacturer’s instructions. The intra-assay coefficients of variance for both the fish were < 12%. The VTG concentration was measured with a Varioskan Flash spectrophotometer (Thermo Fisher Scientific, Tewksbury, MA, USA) at 420 nm, and was expressed as μg/mL.

### Haemolytic complement activity

The activity of the alternative complement pathway hemolytic activity (ACH) was conducted according to the classical method established in fish, with slight modification (e.g. buffer volume, employed basic instruments) [32]. Since no significant change in complement activity was observed at 6 h after the injections of both chemicals, we extended our analysis to 24 h after the injection. Sheep red blood cells (SRBC; 1.5 × 10^6^ cells; National Institute of Toxicological Research, South Korea) were used as the targets of the assay. Reconstitution of the haemolytic activity of olive flounder serum was measured by incubation of the SRBC (5 μl) in 6% olive flounder serum (25 μl) in 10 mM phenol red-free Hank’s buffer with 5 mM Mg^2+^ and 0.15 NaCl (pH 7.3) in a 96-well plate. The plate was incubated for 90 min at 20°C with gentle shaking. Haemolysis was measured spectrophotometrically with a Varioskan Flash spectrophotometer (Thermo Fisher Scientific, Tewksbury, MA, USA) at 414 nm. Complete (100%) and no haemolysis (0%) were measured by adding the washed SRBC (25 μl) to distilled water (100 μl) and phenol red-free Hank’s buffer, respectively. The ACH was calculated as the reciprocal of the serum dilution, causing 50% lysis of SRBC (ACH_50_; U/ml) based on the value of Y/1 − Y against the reciprocal of the serum dilutions on a log–log scaled graph.

### Statistical analysis

Data are presented as mean ± standard deviation (S.D.). We performed a one-way analysis of variance (ANOVA) followed by Duncan’s test to identify differences between the experimental groups. All analyses were performed using SPSS ver. 17.0 (SPSS Inc., Chicago, IL).

## Results

### Sequencing and assembly

To compare the molecular response to different chemical characteristics in the olive flounder, we conducted an RNA-seq based investigation of transcriptional changes in liver tissues exposed to BPS or BaP for 6 h. Three libraries for each chemical and two libraries for the control group were subjected to Illumina RNA seq. The sequenced liver cDNA libraries contained a large number of raw reads, at 62 to 83 million reads per library (S1 Table). After the trimming process, 62–82 million reads were retained from each library.

The *de novo* assembly generated 124,125 transcripts for the liver tissue of *P*. *olivaceus* (Table 1). The lengths of total transcripts ranged from 201 to 17,656 bp with an average length of 984 bp. The overall GC ratio of total transcripts was 47% and the N50 value of those transcripts was 2,110 bp. By employing a bioinformatics platform, 38,909 unigenes were filtered out from the total transcripts (S2 Table). Gene annotation of whole unigenes was performed by BLASTx analysis with the reciprocal BLAST best-hit method (E-value < 1 × 10–10) using NCBI nr protein database. Of unigenes, 28,919 (74%) and 29,979 (77%) transcripts were annotated with GO and KO identity, respectively.

**Table 1:** Summary of the assembly statistic information.

Sample	Merge	
	All transcript contigs	Only longest isoform per gene
Total trinity 'genes'	99,988	99,988
Total trinity transcripts	124,125	99,988
%GC	46.67	46.06
N90 (bp)	335	282
N80	602	420
N70	1,039	661
N60	1,569	1,072
N50	2,110	1,629
N40	2,680	2,237
N30	3,341	2,948
N20	4,172	3,827
N10	5,543	5,228
Maximum contig length (bp)	17,656	17,656
Minimum contig length (bp)	201	201
Median contig length (bp)	431	364
Average contig length (bp)	983.63	789.82
Total assembled bases (bp)	122,093,418	78,972,560

### mRNA expression profiling

**Fig 1:**
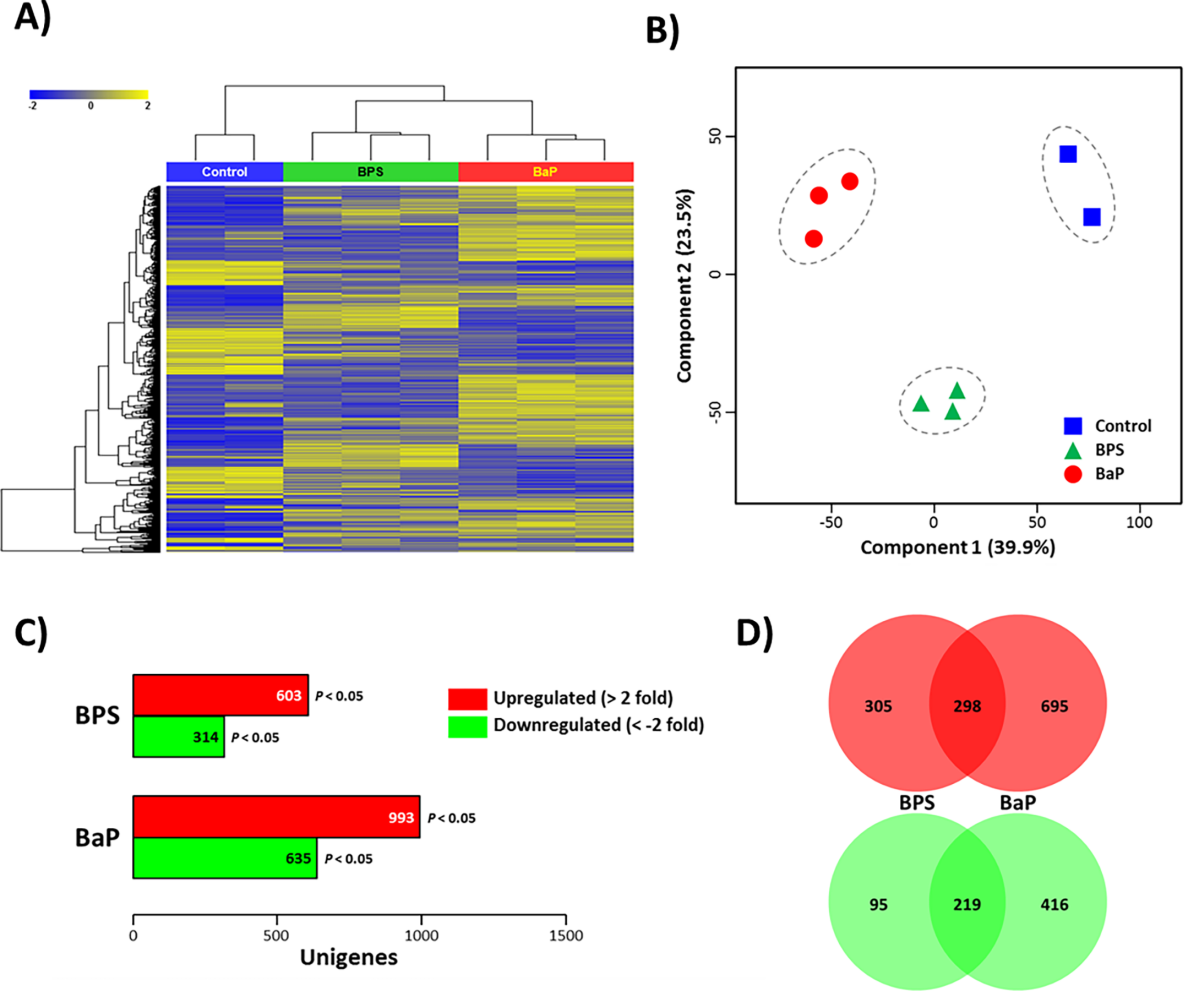
Comparison of transcriptional expression patterns of whole libraries and analysis of chemical-specifically expressed genes. (A) Transcriptional pattern analysis of each library (i.e., two control, three BPS-exposed, and three BaP-exposed liver tissues) by employing heat map and hierarchical clustering; (B) PCA plot analysis of transcriptional profile of each library. Each sample is depicted with a different color. (C) Number of statistically significant transcripts (i.e., over 2 fold; *P* < 0.05) in *P*. *olivaceus* liver tissues exposed to BPS or BaP; (D) the number of uniquely or commonly up- or downregulated transcripts in the *P*. *olivaceus* liver tissues exposed to BPS or BaP. Detailed list of the commonly modulated genes is included in S5 and S6 Tables.

Each library of the three groups (i.e. control, BPS exposure, BaP exposure) was individually aligned to the assembled reference transcriptome in order to compare differentially expressed transcripts. Overall mRNA expressions analyzed by both hierarchical clustering and multidimensional scaling (MDS) plot revealed that those of all three groups separated clearly (Fig 1, S1 Fig). No outlier library was observed in each group, and relatively similar expression profiles were found with hierarchical clustering (Fig 1A). To investigate whether clear differences existed between the control and treated groups, we performed a principle component analysis (PCA), to provide a general overview (Fig 1B). All members involved in each group closely clustered together. While the two control libraries had a relatively higher data variance regarding component two, the scatter was very low regarding the more indicative component one (39.9%). Taken together, both analyses revealed similar relative expression levels for all members analyzed in each group.

A total of 917 unique genes showed significantly different mRNA expression between control and BPS-exposed fish with the criteria > ± 2-fold change and P < 0.05 (603 transcripts upregulated; 314 transcripts downregulated), while 1,628 transcripts showed significant differences in the BaP-exposed fish compared to the control (993 transcripts upregulated; 635 transcripts downregulated) (Fig 1C).

Of upregulated transcripts, 305 mRNAs of BPS- and 695 mRNAs of BaP-exposed fish were chemical-preferentially expressed, whereas 298 mRNAs were commonly upregulated in both chemical exposures (Fig 1D). In the case of downregulated transcripts, 219 mRNAs were common to both chemicals, while 95 and 416 mRNAs were unique to BPS and BaP, respectively.

### Analysis of gene ontology (GO) and KEGG pathway

**Fig 2:**
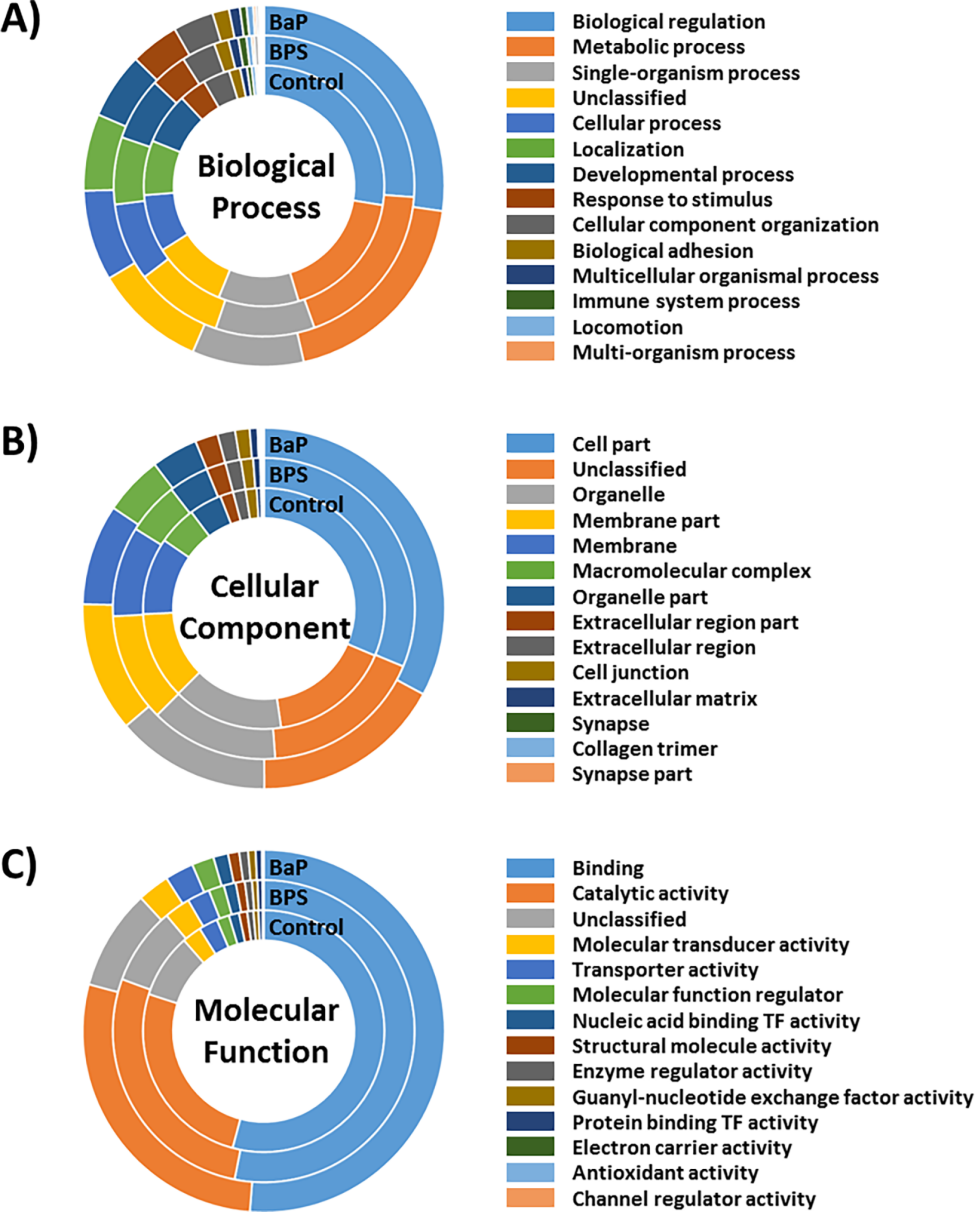
Functional classification of differentially expressed genes. Comparison of Gene Ontology (GO) in terms of (A) biological process, (B) cellular components, and (C) molecular function that were enriched in the BPS- or BaP-exposed liver tissues of *P*. *olivaceus*. Composition of each GO term is represented as a percentage. For detailed information see in S3 Table.

Global functionality of the BPS- and BaP-exposed liver transcriptomes compared to the control group was analyzed by both GO analysis and KEGG pathway mapping. Transcripts were categorized according to their functions within each GO class, such as biological process, cellular process, or molecular function (Fig 2, S3 Table). Overall GO composition was analyzed and the majority was similar among transcriptomes. In the biological process class, the numbers with the GO term “Metabolic process” were increased in the BPS- (2,154) and BaP-exposed liver (2,297) compared that in the control (1,949). Similarly, counts for the GO term “Response to stimulus” were higher in the BPS- (461) and BaP-exposed liver (520) compared to the number in the control (401). Interestingly, the numbers of most GO terms involved in the molecular function class were higher in the BPS- and BaP-exposed liver transcriptomes than those of the control. However, the number of genes categorized in each function of the BPS-exposed liver transcriptome was higher than that from BaP for most GO terms of the three major categories.

**Fig 3:**
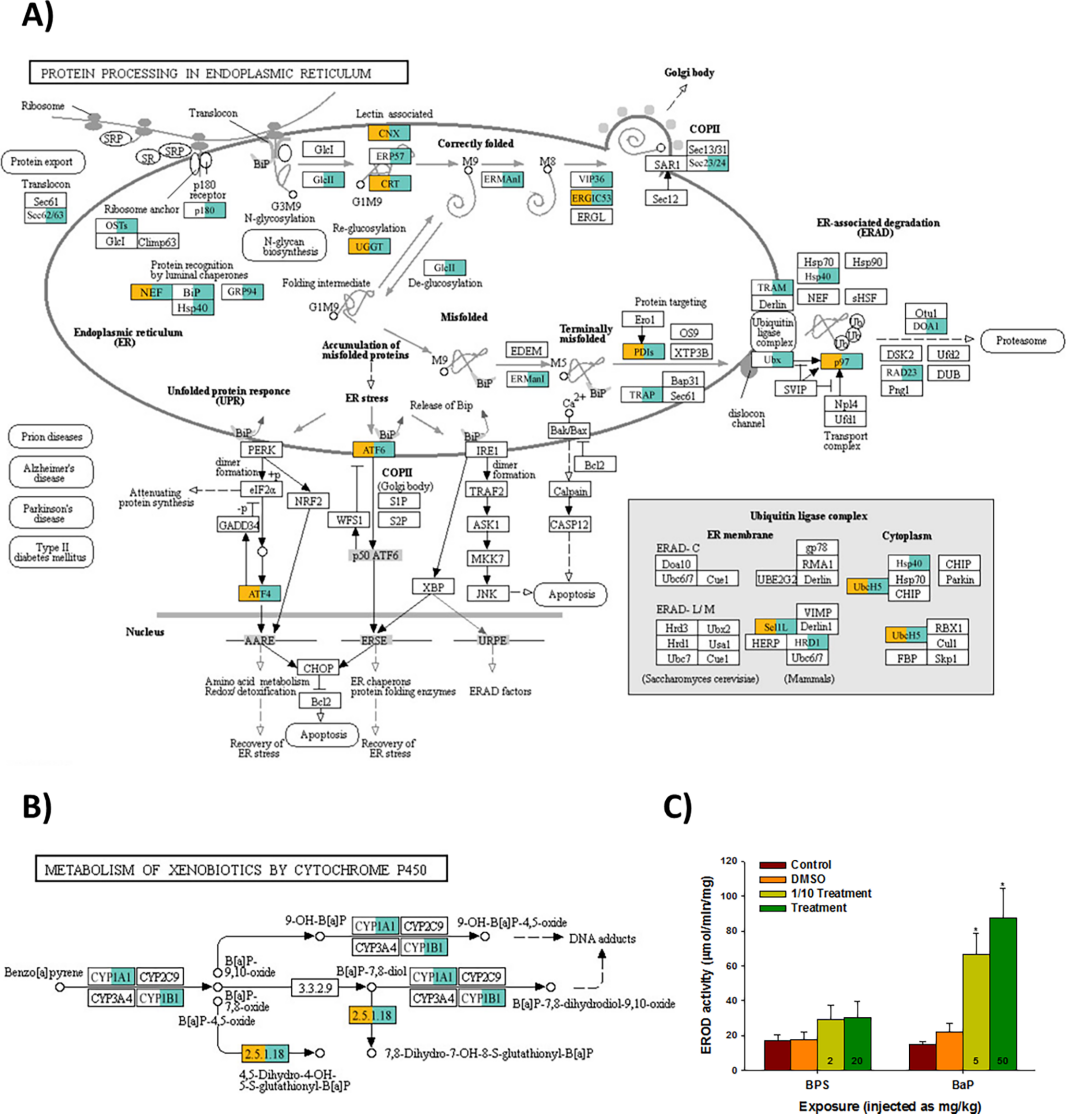
Comparison of KEGG pathways with mapped genes. (A) “Protein procession in endoplasmic reticulum” and (B) Benzo[*a*]pyrene metabolism in “Metabolism of xenobiotics by cytochrome P450” in the BPS- or BaP-exposed liver tissues of *P*. *olivaceus*. The orange color denotes the gene mapped from the BPS-exposed liver transcriptome to each KEGG pathway. The blue-green color denotes the gene mapped from the BaP-exposed liver transcriptomes to each KEGG pathway. Notably, no gene was solely mapped from the BPS-exposed liver transcriptome to a KEGG pathway. (C) The hepatic EROD activity in the liver tissue of BPS- or BaP-injected olive flounder. Data are presented as the mean ± standard deviation (S.D.). Significant differences from the control value are indicated by an asterisk (*) on the data bar (*P* < 0.05).

To test the distinct sensitivity and further usefulness of hepatic transcriptome profiling between the BPS and BaP exposures, all the assembled reads were assigned on the functional classification of KEGG (Table 2 and S4 Table). In both transcriptomes, a high number of transcripts had annotations related to “Metabolic pathways” and “Biosynthesis of secondary metabolites”. Overall KEGG classification showed similar distributions and compositions between transcriptomes. To analyze whether the gene composition matching certain pathways is chemical-specific, we selected a pathway which showed a distinct difference in gene number, and compared each gene’s involvement. Although the pathway, entitled “Metabolic pathways”, showed the largest matched gene number, we were not able to adopt it, as numerous pathways and sub-pathways are involved in the KEGG classification. Among the classifications, the “Protein processing in endoplasmic reticulum” pathway showed different matched genes, as 34 genes of BaP transcriptome were matched to the pathway, while only 12 genes from BPS transcriptome were incorporated in the pathway (Fig 3A).

**Table 2:** Most highly represented KEGG classifications in the liver transcriptomes of the olive flounder *Paralichthys olivaceus* upon BPS and BaP exposures.

BPS exposure			BaP exposure		
Classification	Map ID	#	Classification	Map ID	#
Metabolic pathways	1100	77	Metabolic pathways	1100	129
Biosynthesis of secondary metabolites	1110	28	Biosynthesis of secondary metabolites	1110	39
Microbial metabolism in diverse environments	1120	23	Protein processing in endoplasmic reticulum	4141	34
Biosynthesis of antibiotics	1130	20	Biosynthesis of antibiotics	1130	28
Pathways in cancer	5200	15	Pathways in cancer	5200	27
Carbon metabolism	1200	15	Microbial metabolism in diverse environments	1120	24
Protein processing in endoplasmic reticulum	4141	13	PI3K-Akt signaling pathway	4151	19
PI3K-Akt signaling pathway	4151	13	Carbon metabolism	1200	18
Non-alcoholic fatty liver disease (NAFLD)	4932	12	Epstein-Barr virus infection	5169	18
Human papillomavirus infection	5165	12	FoxO signaling pathway	4068	16

Since the pathway of “Metabolism of benzo[*a*]pyrene by cytochrome P450” is registered in the KEGG database, we additionally checked the number and composition of matched genes to the pathway. As shown in Fig 3B, CYP1A-involved metabolism genes were highly expressed in the BaP-exposed liver transcriptome. In addition, significant increases in EROD activity were observed at 5 and 50 mg/kg bw of BaP injection into the liver tissue, while no significant change was measured in the BPS-exposed olive flounder (Fig 3C). The signaling, BaP-mediated CYP1A expression, is not uncommon in teleosts, but comparison of the mated gene clearly showed that the flounder hepatic transcriptome is differentially sensitive to specific chemicals; this profiling will be useful for monitoring marine contaminants.

### Comparison of the BPS- and BaP-exposed liver transcriptomes

**Fig 4:**
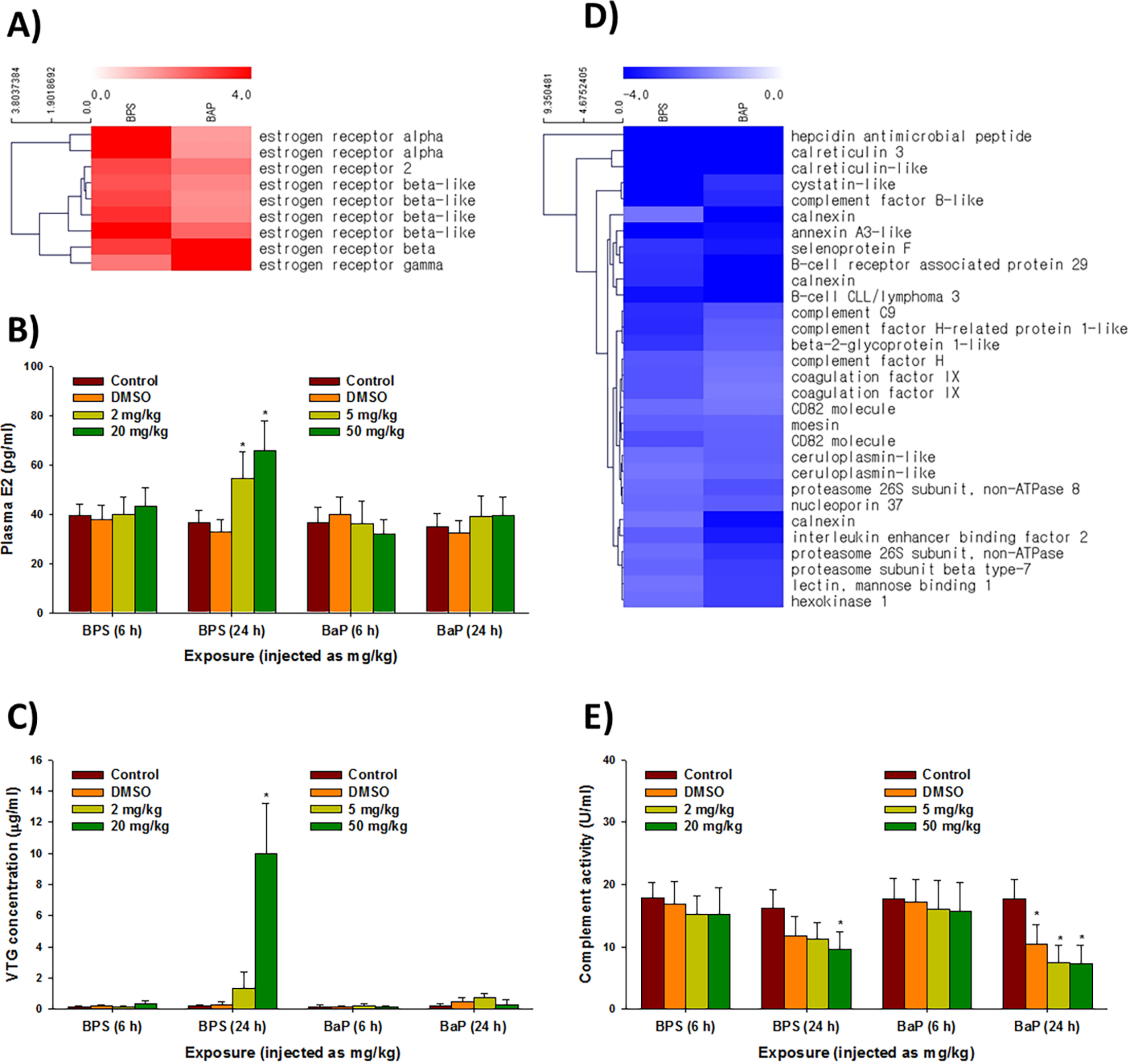
Hierarchical clustering analysis of differentially expressed genes and biochemical evidences. (A) Transcriptional profiles of estrogen receptor genes (i.e. *erα*, *erβ*, and *erγ*) in the BPS- or BaP-exposed liver tissues of *P*. *olivaceus*; (B) the effect of BPS and BaP injections on the plasma 17β-estradiol (E2) concentration in the liver tissues of *P*. *olivaceus*. E2 concentration is expressed as pg/mL. Each value is an average of five biological replicates, and data are shown as means ± S.D; (C) the effect of BPS and BaP injections on the plasma vitellogenin (VTG) concentration in the liver tissues of *P*. *olivaceus*. VTG concentration is expressed as μg/mL. Each value is an average of five biological replicates, and data are shown as means ± S.D; (D) transcriptional expressions of genes associated with the innate immunity in the BPS- or BaP-exposed liver tissues of *P*. *olivaceus*. (E) the effect of BPS and BaP injections on the plasma complement activity in the liver tissues of *P*. *olivaceus*. The activity is expressed as U/mL. Each value is an average of five biological replicates, and data are shown as means ± S.D. The asterisk symbol (*) indicates statistical significance (*P* < 0.05) compared to the control values.

A comparative analysis of the *P*. *olivaceus* liver transcriptomes was performed to identify transcripts conserved in the liver tissue and those unique to each chemical exposure. Table 3 and Table 4 show the 20 most highly up- and downregulated genes in the BPS- or BaP-exposed liver tissue, respectively. Overall, 4 of the 20 most highly upregulated genes in the BPS-exposed liver transcriptome were related to egg process and vitellogenesis, such as vitellogenin 1 (*vtga*), 2 (*vtgb*), and zona pellucida sperm-binding protein (*zp3* and *zp4*) (Table 3). Since the transcription of *vtg* genes and synthesis of the VTG protein are strongly associated with estrogen level, we further analyzed estrogen-related transcriptional and physiological responses. In the increased genes, we identified that the estrogen receptor (ER) family such as *erα* and *erβ* genes were significantly increased compared to their expression in BaP-exposed liver tissue (Fig 4A). To confirm the potential correlation between upregulation of the *vtg* and *er* genes, we measured the plasma E2 and VTG concentrations in the serum samples after BPS or BaP injection. As a result, significant increases in plasma E2 were observed after 24 h in the BPS-injected olive flounder (*P* < 0.05), while no significant change was measured in the BaP treatment (*p* > 0.05) (Fig 4B). A significant elevation in the plasma VTG was also observed only after 24 h in the BPS-injected olive flounder (*P* < 0.05) (Fig 4C).

**Table 3:** Differentially expressed mRNAs in BPS/control [fold change (log 2) >10].

Gene symbol	Fold Change	Up or Down	KEGG orthology
MFSD2AB	54.69	up	lco:104920439 mfsd2a; major facilitator superfamily domain containing 2A
VTG1	45.45	up	lco:104931934 VgA; vitellogenin-1-like
-	42.91	up	unnamed protein product
VTG2	28.09	up	lco:104931936 VgB; vitellogenin-2-like
GPT2L	24.62	up	lco:104929222 alanine aminotransferase 2-like
IGFBP1A	23.65	up	lco:104929331 insulin-like growth factor-binding protein 1
ZP3	23.30	up	lco:104940476 zona pellucida sperm-binding protein 3
MIOX	19.20	up	lco:104918107 miox; myo-inositol oxygenase
FAM46BA	18.23	up	lco:104938655 fam46b; protein FAM46A
IGFBP1A	17.75	up	lco:104929331 insulin-like growth factor-binding protein 1
CCER	15.81	up	lco:104930694 coiled-coil domain-containing glutamate-rich protein 1-like
ALDOB	14.58	up	lco:104939510 aldob; fructose-bisphosphate aldolase B
AGXTB	14.11	up	mze:101480149 serine—pyruvate aminotransferase-like
FAM20C	13.83	up	mze:101477115 extracellular serine/threonine protein kinase FAM20C-like
MUC2	13.19	up	lco:104921854 prg4; proteoglycan 4
UNNAMED	12.73	up	lco:109140524 alanine aminotransferase 2-like
SI:DKEY-178E17.3	12.58	up	lco:104921667 somatomedin-B and thrombospondin type-1 domain-containing protein-like
MOB2	11.62	up	lco:104918109 MOB kinase activator 2
ZP4	11.50	up	lco:104940483 zona pellucida sperm-binding protein 4-like
ZGC:112285	11.36	up	mze:101487569 elastase-1-like
GDF15	-99.77	down	lco:104920968 gdf15; growth differentiation factor 15
OSER1	-32.97	down	lco:104938359 oser1; oxidative stress responsive serine rich 1
DIO3	-24.48	down	lco:104924692 thyroxine 5-deiodinase-like
ANGPTL4	-22.35	down	lco:109141011 angiopoietin-related protein 4-like
PHGDH	-19.82	down	lco:104931034 phgdh; phosphoglycerate dehydrogenase
TNIP3	-18.58	down	lco:104933799 TNFAIP3-interacting protein 1-like
PHGDH	-15.40	down	lco:104931034 phgdh; phosphoglycerate dehydrogenase
TNNT1	-15.27	down	lco:104939292 troponin T, slow skeletal muscle-like
-	-13.82	down	unnamed protein product
HAMP	-11.89	down	xma:102230476 hamp; hepcidin antimicrobial peptide
TGM2L	-11.40	down	lco:104929140 protein-glutamine gamma-glutamyltransferase 2-like
-	-10.38	down	unnamed protein product
CALCOCO1B	-8.92	down	mze:101469246 calcium-binding and coiled-coil domain-containing protein 1-like
KRT18	-8.53	down	lco:104930767 keratin, type I cytoskeletal 18-like
SERPINH1B	-8.49	down	ola:100529194 serpinh1; serpin H1 isoform X1
HP	-8.34	down	mze:101466498 haptoglobin-like
GK	-8.13	down	lco:104922970 gck; glucokinase
ISYNA1	-7.62	down	lco:104926597 isyna1; inositol-3-phosphate synthase 1-A
TCNL	-7.61	down	lco:104937502 transcobalamin-1
IGFBP3	-7.55	down	lco:104927720 igfbp3; insulin like growth factor binding protein 3

**Table 4:** Differentially expressed mRNAs in BaP/control [fold change (log 2) >10].

Gene symbol	Fold Change	Up or Down	KEGG orthology
CYP1A	176.14	up	lco:104920743 CYP1A; cytochrome P450 1A1
UGT1A1	60.52	up	lco:109136658 UDP-glucuronosyltransferase-like
IGFBP1A	42.85	up	lco:104929331 insulin-like growth factor-binding protein 1
-	29.24	up	tng:GSTEN00019789G001 unnamed protein product
PDK2B	29.16	up	lco:104927162 pyruvate dehydrogenase (acetyl-transferring) kinase isozyme 2, mitochondrial-like
GM46320	22.65	up	mmu:108167963 Gm46320; predicted gene, 46320
MIOX	21.93	up	lco:104918107 miox; myo-inositol oxygenase
GPT2L	21.88	up	lco:104929222 alanine aminotransferase 2-like
A4GUE9	16.28	up	lco:109140778 insulin-like growth factor II
MOB2	16.19	up	lco:104918109 MOB kinase activator 2
ZGC	15.50	up	mze:101487569 elastase-1-like
GPT2-LIKE	13.44	up	lco:109140524 alanine aminotransferase 2-like
TIPARP	13.28	up	lco:104924110 tiparp; TCDD inducible poly (ADP-ribose) polymerase
WFDC2	12.18	up	lco:104919425 WAP four-disulfide core domain protein 18-like
CYP26A1	12.14	up	lco:104928862 cytochrome P450 26A1
-	12.10	up	nle:105739288 uncharacterized LOC105739288
SI:DKEY-188C14	12.08	up	lco:104923163 serine/arginine repetitive matrix protein 1-like
EEF2B	11.73	up	mze:101482186 elongation factor 2
SI:DKEY-188C14	11.72	up	lco:104923163 serine/arginine repetitive matrix protein 1-like
AGXTB	11.63	up	mze:101480149 serine—pyruvate aminotransferase-like
GDF15	-344.78	down	lco:104920968 gdf15; growth differentiation factor 15
TNIP3	-66.63	down	lco:104933799 TNFAIP3-interacting protein 1-like
HSPA5	-34.02	down	lco:104925213 hspa5; 78 kDa glucose-regulated protein
ANGPTL4	-32.22	down	lco:109141011 angiopoietin-related protein 4-like
PFKFB3	-27.26	down	lco:104933238 pfkfb3; 6-phosphofructo-2-kinase/fructose-2,6-biphosphatase 3
PHGDH	-23.58	down	lco:104931034 phgdh; phosphoglycerate dehydrogenase
OSER1	-22.25	down	lco:104938359 oser1; oxidative stress responsive serine rich 1
HSP90B1	-18.65	down	mze:101467243 hsp90b1; heat shock protein 90 beta family member 1
CRELD2	-17.52	down	lco:104926574 creld2; cysteine rich with EGF like domains 2
MIDN	-17.09	down	lco:104924845 midnolin-A-like
SGK1	-13.80	down	lco:104925665 sgk1; serum/glucocorticoid regulated kinase 1
TGM2L	-13.53	down	lco:104929140 protein-glutamine gamma-glutamyltransferase 2-like
IARS	-12.37	down	lco:104931396 iars; isoleucyl-tRNA synthetase
LIPG	-11.84	down	lco:104922983 lipg; lipase G, endothelial type
CALCOCO1B	-11.17	down	mze:101469246 calcium-binding and coiled-coil domain-containing protein 1-like
HAMP	-11.12	down	xma:102230476 hamp; hepcidin antimicrobial peptide
DNAJB11	-10.99	down	mze:101474382 dnajb11; DnaJ heat shock protein family (Hsp40) member B11
YARS	-10.93	down	lco:104920437 yars; tyrosyl-tRNA synthetase
KRT18	-9.80	down	lco:104930767 keratin, type I cytoskeletal 18-like
CALR3B	-9.53	down	lco:104931427 calreticulin-like

**Fig 5:**
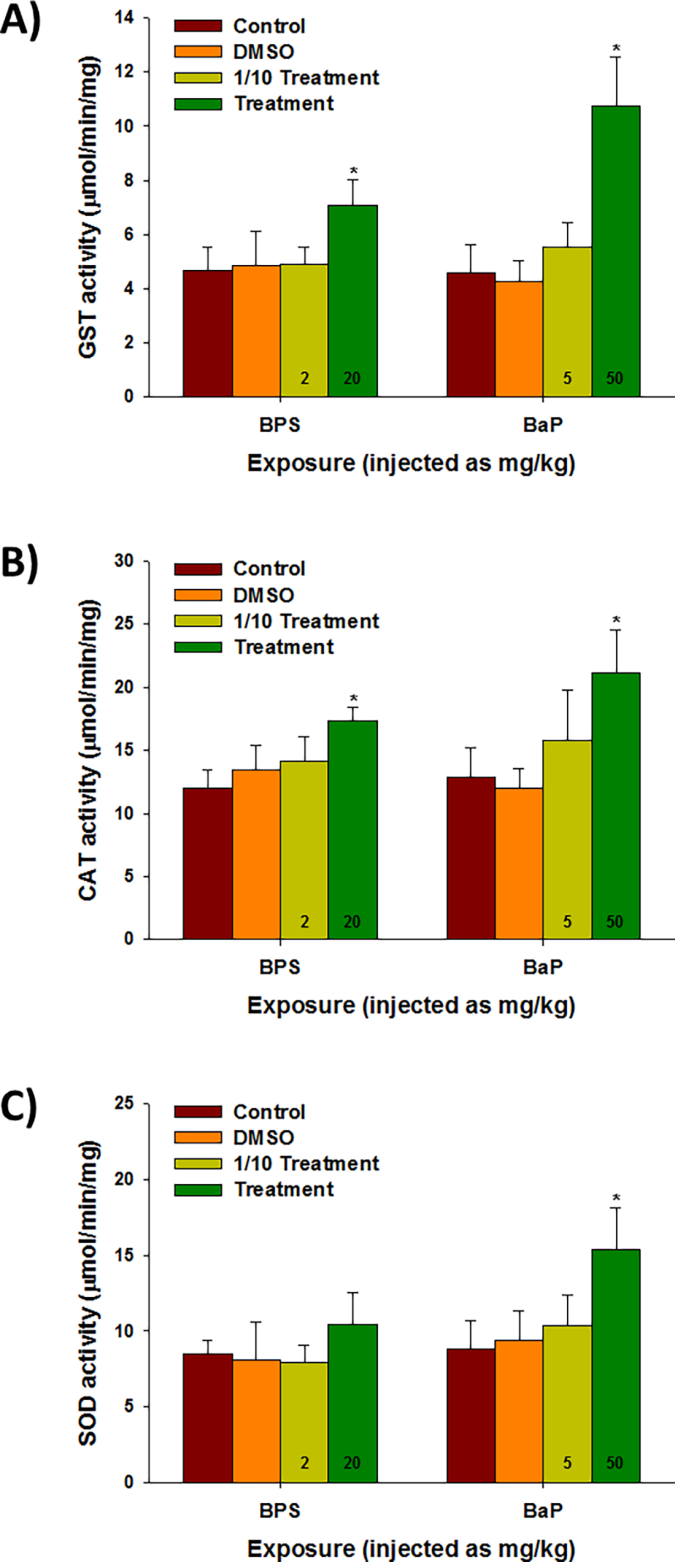
Enzymatic activities of biotransformation and antioxidant defense systems. The effect of the BPS and BaP injections on the enzymatic activities of the (A) GST, (B) CAT, and (C) SOD proteins in the liver tissues of *P*. *olivaceus* at 6 h. The activity is expressed as μmol /min/mL. Each value is an average of five biological replicates, and data are shown as means ± S.D. The asterisk symbol (*) indicates statistical significance (*P* < 0.05) compared to the control values.

In the case of the BaP-exposed liver, genes involved in drug/xenobiotic metabolism were highly upregulated, as the most highly expressed gene was *cyp1a1*, with 176 a fold change, followed by UDP-glucuronosyltransferase (*ugt1a1*) (Table 4). We observed that 10 genes related to drug/xenobiotic metabolism and the antioxidant defense system (i.e. UDP-glucuronosyltransferase, *ugt2A1*; glutathione S-transferase, *gst*; microsomal GST, *mgst*; GST zeta, *gstz*; and catalase, *cat*) were commonly upregulated by BPS and BaP exposures (S5 Table). The hepatic enzymatic activity of the total GST protein was significantly elevated by the injections of both chemical (*P* < 0.05) (Fig 5A). Additionally, the CAT activity showed significant increases in its response to both chemicals (*P* < 0.05) (Fig 5B), while only BaP increased the SOD activity at 6 h after the injection (*P* < 0.05) (Fig 5C).

Notably, the CYP1A1 and UGT1A proteins are members of the “aryl hydrocarbon receptor (AhR) gene battery” which comprises several important metabolizing enzymes for biotransformation and detoxification of xenobiotics and toxicants [33]. Two-contigs coding for the *ahr* gene were significantly upregulated by BaP exposure (c41830_g1_i3: 2.33-fold upregulated, P = 0.004; c41830_g1_i4: 3.07-fold upregulated, P = 0.009), while their modulations were not significant following BPS exposure. Transcriptional expressions of additional “AhR gene battery” members were also significantly increased such as *cyp1b1* (c47411_g3_i2: 2.40-fold upregulated, P = 0.029; c47411_g3_i3: 2.31-fold upregulated, P = 0.031; c47411_g3_i4: 2.94-fold upregulated, P = 0.014) and *gsta* (c40603_g1_i1: 2.11-fold upregulated; P = 0.021).

Although numerous genes associated with diverse pathways were downregulated by BPS or BaP exposure, mRNA expressions of several genes involved in the innate immunity including complement genes were decreased by both BPS and BaP exposure (Fig 4D, S6 Table). To further assess the potential correlation between the decrease in mRNA expression and protein activity, we measured the haemolytic complement activity of olive flounder serum. The plasma haemolytic complement activity of BPS- or BaP-exposed olive flounder was decreased, but only reached a statistically significant level after 24 h (*P* < 0.05) (Fig 4E). In this experiment, the DMSO also decreased the activity after 24 h (*P* < 0.05, observed in the plasma of BaP-injected olive flounder).

### qPCR validation

**Fig 6:**
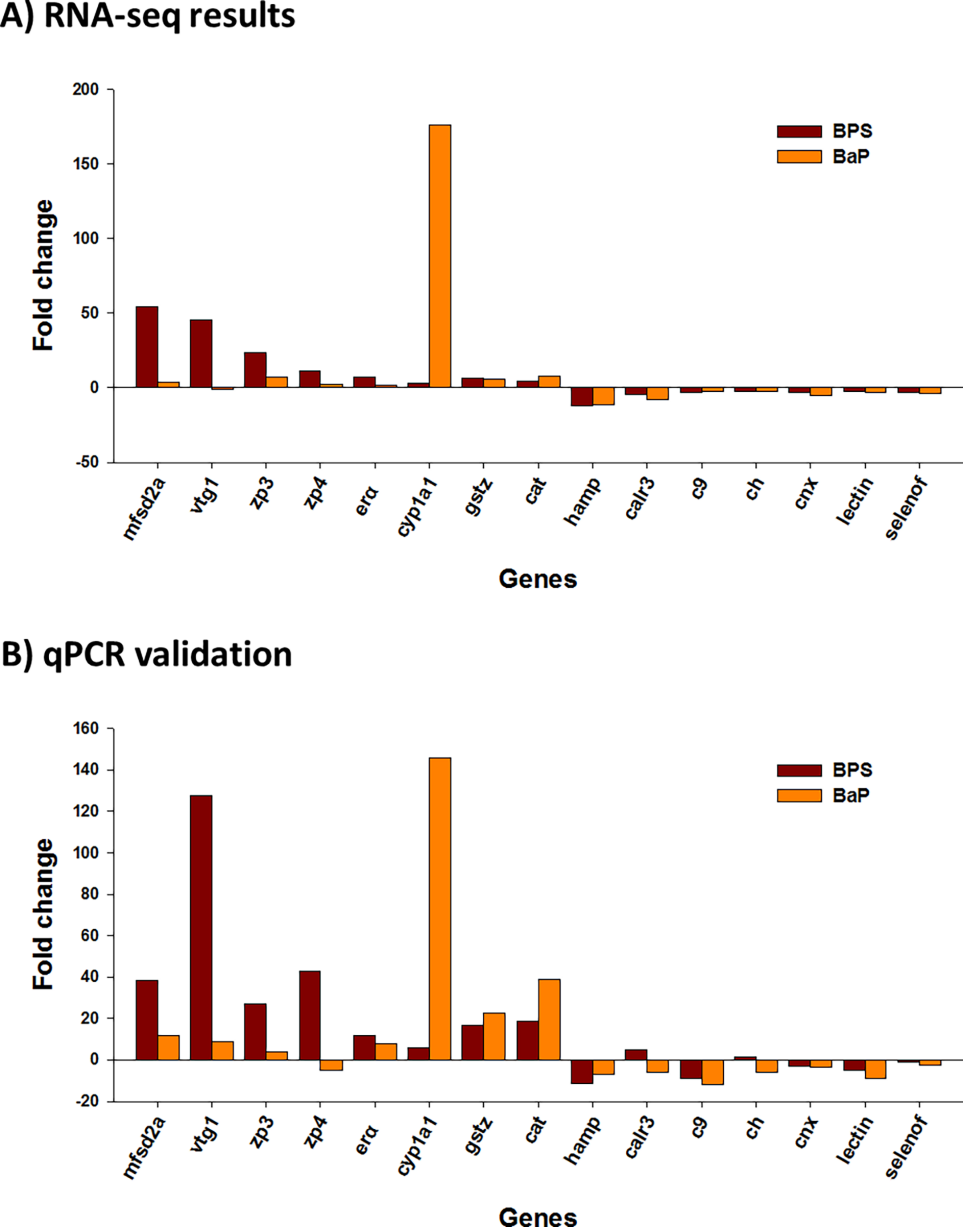
qPCR validation results on the mRNA expressions of 15 randomly selected genes. (A) The mRNA expressions of 15 genes were selected from the RNA-seq data (*P* < 0.05). (B) Validation of the mRNA expression patterns of the selected 15 genes by qPCR. Abbreviations of the gene names are as follows: Major facilitator superfamily domain contating 2A, *mfsd2a*; Vitellogenin 1, *vtg1*; Zona pellucida sperm-binding protein 3, *zp3*; Zona pellucida sperm-binding protein 4, *zp4*; Estrogen receptor alpha, *erα*; Cytochrome P450 1A1, *cyp1a1*; Glutathione S-transferase zeta 1, *gstz*; Catalase, *cat*; Hepcidin antimicrobial peptide, *hamp*; Calreticulin 3, *calr3*; Complement C9, *c9*; Complement H, *ch*; Calnexin, *cnx*; *lectin*; Selenoprotein F, *selenof*.

To verify differentially expressed genes for each chemical, mRNA expressions of highly represented genes were analyzed by real-time qRT-PCR (Fig 6). The details on primer information are shown in S7 Table. Quantitative expressions of 15 randomly selected genes among the highly up- or downregulated genes in BPS- or BaP-exposed liver tissue showed the high reproducibility of the biological replicates. Most genes showed similar mRNA expression patterns between the two platforms, RNA-seq and qPCR, except for *calr3* and *ch* in the BPS-injected sample, and *vtg1* and *zp4* in the BaP-injected sample.

## Discussion

Overall comparison of GO compositions and their ratio among transcriptomes indicates that both chemicals have the potential to induce or inhibit transcriptional expression, and the modulated genes may be associated with cellular homeostasis in liver tissue. This result further implies that diverse pathways are active in the liver tissue of *P*. *olivaceus*, some of which have chemical-specific roles. KEGG analysis revealed that a high number of transcripts were associated with general metabolism and metabolite processes in both transcriptomes. A possible explanation for this could be that the fish liver tissue employs numerous enzymes and functional systems to maintain homeostasis by converting molecules (e.g., drug/xenobiotics metabolism) and regulating energy balance (e.g., glycogenesis, gluconeogenesis) [34]. Both chemicals used in this study are detoxified and excreted by metabolism in the fish liver [35–39]. BPS was biotransformed into a variety of gluco- and sulfo-conjugated metabolites in zebrafish models (primary hepatocytes, ZFL, and ZELH-zfER cell lines) [40]. BaP-diones accounted for the majority of metabolites in the liver tissue of two species of Ictalurid catfish [41]. In fact, increase or decrease in the transcriptome after exposure to exogenous chemicals is not surprising as this phenomenon has been continuously observed in diverse aquatic animals, including fish. However, it is notable that genes mapped to a KEGG pathway by comparative transcriptome analysis showed chemical-specific patterns. The results clearly suggest that transcriptome profiling and transcriptional sensitivity in the *P*. *olivaceus* liver tissue is promising as a strong biomarker with distinct characteristics on exposure to certain chemicals.

Vitellogenin (Vtg), as the precursor of egg yolk protein, is important for oocyte development and embryogenesis in oviparous oogenesis, as Vtg is secreted into the bloodstream and incorporated into the growing oocytes [42]. Estrogen and other hormones mainly control the synthesis of Vtg protein, and its extra-hepatic expressions have been detected in teleost in addition to its major production in the liver tissue [43]. Induction of Vtg is known to be a strong biomarker for estrogenic compounds, such as endocrine disrupting chemicals (EDCs) in fish [44–46]. Induction of Vtg has also been observed in immature fish (e.g., larvae and juveniles) [47], as shown in the present study. Although BPS has been considered as an alternative of BPA, its estrogenic potential and adverse effects (e.g., endocrine-disrupting effects) on the physiological functions have been consistently reported in vertebrates, including fish [17, 48, 49]. Developmental exposure to BPS significantly increased plasma Vtg levels in both male and female zebrafish [50]. In fish, vitellogenin production and vitellogenesis are mainly mediated through the induction of ERs [51, 52]. In fact, BPS can directly bind to the ERs in a concentration-, tissue-, or species-specific manner [53–56]. Based on the transcriptional increases of the ER family and plasma E2 level observed in this species, we would conclude that BPS injection triggered vitellogenic responses by exerting estrogenic effects in *P*. *olivaceus*.

Zona pellucida proteins (ZPs) share a conserved ZP domain and are constituents of the egg chorion under estrogenic control in fish [57]. Although the potential function of ZPs in the recognition and interaction between egg and sperm has been rarely studied in fish, it is notable that xenoestrogen treatments strongly induced genes associated with fish zonagenesis [42, 57]. In addition, mRNA expressions of ZPs (i.e., *zp3*, *zpb*, *zpc*) were significantly increased by the estrogen mimic octylphenol and nonylphenol exposure in the Atlantic salmon smolts and cichlid fish [58, 59]. Thus, transcriptional expression of *P*. *olivaceus* ZPs can be assertive molecular indicators for xenoestrogens.

Besides the well-characterized carcinogenicity and genotoxicity of BaP, previous studies have suggested that BaP is biotransformed to epoxides and quinones by drug/xenobiotic metabolism via activation of the “AhR gene battery” [18, 33, 60, 61]. In *P*. *olivaceus*, BaP injection strongly increased mRNA expression of the members of “AhR gene battery” in a mechanistic manner through phase I (e.g. *cyp1a1*) to phase II (e.g. *ugt2A1*, *gsts*) drug/xenobiotic metabolism with inductions of the EROD and GST activity. The AhR-mediated process was originally characterized as a regulator of PAHs such as BaP beyond the well-characterized TCDD-induced toxicity [62]. In addition to the classical AhR-mediated mechanism, induction of the *cyp1a* gene at both transcriptional and translational levels has been highlighted as one of the major biomarkers for BaP exposure in numerous teleost [63–67]. Several fish also showed BaP-triggered inductions of members of the UGT [68, 69] or GST gene families [41, 67, 70, 71]. Some GST proteins are strongly involved in the AhR-mediated process [33, 61]. Taken together, the overall response of the “AhR gene battery” is likely to be sensitive to BaP injection in *P*. *olivaceus*. Although our results are not sufficient to prove actual biotransformation because enzymatic activity and chemical structure of BaP metabolites were not analyzed, a significant elevation of the mRNA expressions with induction of the EROD activity indicates their potential as strong biomarkers and early signals of BaP exposure in *P*. *olivaceus*.

The mRNA expression and enzymatic activity of genes associated with the antioxidant defense system was increased in the liver transcriptome of *P*. *olivaceus* after exposure to both chemicals studied. Over the last ten years, the prooxidant role of BPA has been extensively studied, including BPA-induced intracellular free radicals [e.g. reactive oxygen species (ROS)], oxidative stress, and subsequent DNA damage in numerous *in vitro* models and vertebrates, including fish [72–74]. Although there still needs to be rigorous study to understand BPS-induced oxidative stress, recent research suggests that BPS, as the main BPA substituent, can also promote the generation of ROS and alter antioxidant balance related to oxidative stress in vertebrates [75–77]. Taken together, the results of the present study suggest that BPS-triggered induction of oxidative stress may produce potential pleiotropic effects such as DNA damage, developmental toxicity, reproductive toxicity, and disease beyond its endocrine disrupting properties in fish.

Typically, PAHs, including BaP, are known to act as strong mutagens and/or carcinogens via oxidative stress induction. BaP can generate large amounts of intracellular ROS during the biotransformation process and the modulation of the antioxidant defense system [78]. In detail, its metabolites (e.g. BaP-3-phenol, BaP-quinone) are believed to directly bind to intracellular micro and macromolecules with the formation of ROS [79]. In teleost, studies on transcriptional activation and enzymatic modulation of the antioxidant defense system have consistently highlighted their biomarker potential for BaP exposure prior to the phenotypic and physiological toxic effects [67, 71, 80–83]. Thus, we consider that BaP-triggered oxidative stress may be significant in the liver tissues of *P*. *olivaceus*. Although the example of KEGG classification, “Protein processing in endoplasmic reticulum”, was used for testing different transcriptional sensitivities, the pathway also suggested biological evidence of BaP-triggered oxidative stress, and the subsequent induction of the endoplasmic reticulum-stress pathway, as shown in *in vitro* systems [84, 85]. Since there is very limited information available on ubiquitin ligase (E3) complexes and ubiquitination in teleosts, we were not able to estimate its role in the BaP-specific transcriptional expression observed in this species. Activated AhR induced the expressions of estrogen receptor (ER) target genes, which promoted ER proteolysis through assembling the E3 complex [86]. The BaP-triggered AhR pathway could be associated with the ubiquitination of certain proteins damaged by BaP in the liver tissues of *P*. *olivaceus*.

Another interesting aspect revealed by transcriptome profiling is that mRNA expression of genes involved in the innate immune system were reduced by both BPS and BaP injection in this species. Similarly to the decreased mRNA expressions of several complement genes, the haemolytic complement activity was also significantly decreased by both chemicals. The innate immunity of vertebrates has been considered as one of the targets of BPA [87]. BPA modulates various aspects of the immune system, including innate and adaptive immunity, by altering B cell functions, T cell subsets, dendritic cell, and macrophage biology in aquatic animals [87, 88]. In teleost, both acute and chronic exposure to BPA significantly affect the immune system such as inhibition of transcriptional expressions of genes related to the immune response [e.g., IFNγ, IL1β, IL10, Mx, TNFα, CC-chemokine, CXCL-clc, the Toll-like receptors (TLRs) signaling pathway (TLR3, TRIF, MyD88, SARM, IRAK4, and TRAF6), and NF-κB-associated immune genes], serum immune parameters [e.g. levels of immunoglobulin M (IgM), C-reaction Protein (CRP), and complement component 3] [87–91]. Although BPS assays on the fish immune system have not been extensively undertaken to date, a recent study suggested that immune defense is diminished by exposing parental generations to environmentally relevant concentrations of BPS in zebrafish [92]. Suppressive effects of BaP on immune systems have been extensively reviewed in teleost [93–95]. In particular, the liver tissues contribute to immune surveillance by controlling circulating antigens and innate immune cells [96, 97]. Taken together, modulation of immune transcriptomes would be involved in dysregulation of innate immunity, as the physical and chemical barriers of the innate immune system can control numerous waterborne pathogens in fish.

**Fig 7:**
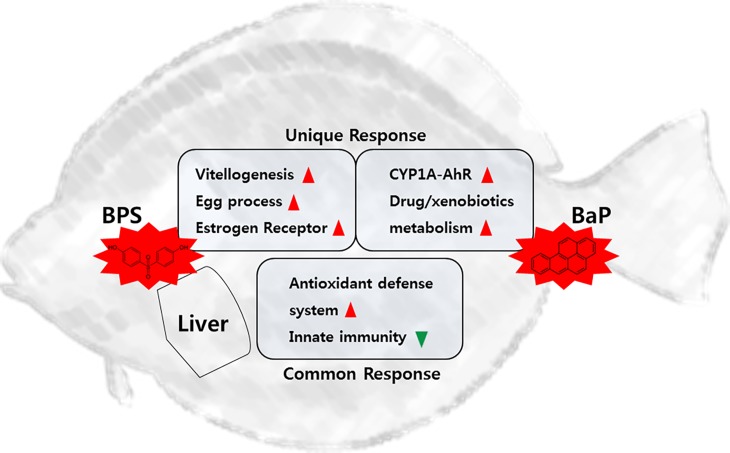
Schematic summary of unique and common transcriptional responses in the BPS- and BaP-exposed liver tissues of *P*. *olivaceus*. Red arrow means increased transcriptional metabolism and green arrow represents decreased transcriptional metabolism.

The aim of this study was to elucidate the potential advantages, particularly in monitoring of coastal regions and in transcriptional profiling of the *P*. *olivaceus* liver tissue. Finally, we propose a schematic diagram for transcriptional responses involved in the spectrum of flatfish (i.e., chemical-specific) or common induction and inhibition of possible mechanisms in the BPS- or BaP-exposed liver tissues of *P*. *olivaceus* with biochemical evidence (Fig 7). Our results show that the application of transcriptome profiling is a promising testing method for the study of exposure to different environmental pollutants, as each transcriptome can respond to certain chemicals via both specific and common response mechanisms.

## Supporting information

S1 Fig Comparison of transcriptional expression patterns of whole libraries. A) Hierarchical clustering analysis of each library (i.e. two control liver tissues, three BPS-exposed liver tissues, and three BaP-exposed liver tissues) by employing heat map and hierarchical clustering. B) Overall transcriptional profile of each library. Similarity is depicted with different colors.
(TIF)

S1 Table Summary of the raw read sequencing information on each replicate. (XLSX)

S2 Table Information on the raw data of mRNA expression, statistics, and annotation of entire contigs. (XLSX)

S3 Table Comparison of gene ontology (GO) between BPS- and BaP-exposed liver transcriptomes. (XLSX)

S4 Table Comparison of KEGG pathways between BPS- and BaP-exposed liver transcriptomes. (XLSX)

S5 Table Information on commonly up-regulated mRNA expressions by BPS and BaP exposures. (XLSX)

S6 Table Information on commonly down-regulated mRNA expressions by BPS and BaP exposures. (XLSX)

S7 Table Primer list used for qPCR validation. (XLSX)
