# Supplementary figures and images for: Comparative analysis of distinctive transcriptome profiles with biochemical evidence in bisphenol S- and benzo[a]pyrene-exposed liver tissues of the olive flounder Paralichthys olivaceus
Source: PLoS One. 2018 May 1;13(5):e0196425. doi: 10.1371/journal.pone.0196425 (PMC5929548; doi:10.1371/journal.pone.0196425)

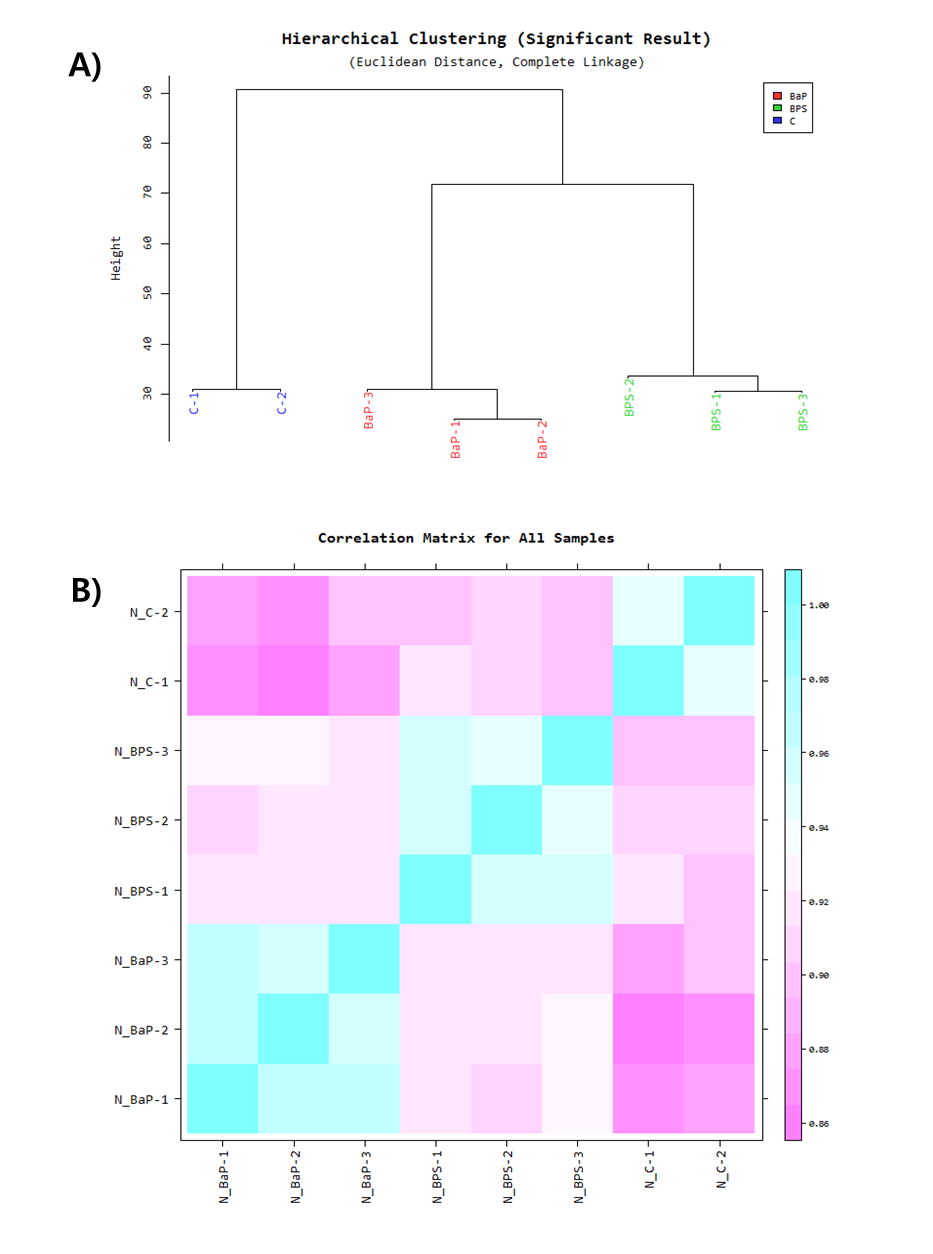

Supplement: S1 Fig — A) Hierarchical clustering analysis of each library (i.e. two control liver tissues, three BPS-exposed liver tissues, and three BaP-exposed liver tissues) by employing heat map and hierarchical clustering. B) Overall transcriptional profile of each library. Similarity is depicted with different colors. (TIF) [file pone.0196425.s001.tif]
